# NMR Assignments of Six Asymmetrical N-Nitrosamine Isomers Determined in an Active Pharmaceutical Ingredient by DFT Calculations

**DOI:** 10.3390/molecules27154749

**Published:** 2022-07-25

**Authors:** Hao-Yue Guan, Yu-Fei Feng, Bai-Hao Sun, Jian-Zhao Niu, Qing-Sheng Zhang

**Affiliations:** Chemical Drugs Control Institute of China National Institutes for Food and Drug Control (NIFDC), No. 2 Tian Tan Xi Li Street, Dong Cheng District, Beijing 100050, China; guanhaoyue@nifdc.org.cn (H.-Y.G.); fengyf@nifdc.org.cn (Y.-F.F.); baihaosun@163.com (B.-H.S.)

**Keywords:** asymmetrical N-nitrosamines, isomers, NMR assignment, density functional theory calculation, variable temperature ^1^H-NMR experiments

## Abstract

N-nitrosamines, which are well-known pro-mutagens, are found in drugs, pickled food and tobacco. Therefore, controlling their concentrations is very important. When an HPLC, GC or NMR analysis is conducted to investigate certain asymmetrical N-nitrosamines, two sets of signals attributed to the asymmetric N-nitrosamine isomers are usually observed. However, few reports on the NMR assignment of asymmetrical N-nitrosamine isomers have been published. In this study, we investigated the NMR assignments of the *Z/E* isomers of six asymmetrical N-nitrosamines by means of density functional theory (DFT) calculations. The configuration of the major isomer of asymmetrical N-nitrosamine **3** was the Z-configuration. The configuration of the major isomers of asymmetrical N-nitrosamines **4**–**7** was the *E*-configuration. Then, we determined the *Z/E* ratios of these asymmetrical N-nitrosamines by means of variable temperature (VT) and room temperature (RT) ^1^H-NMR experiments. The ratios of the *Z/E* isomer **3** quickly increased beyond 100% in the VT ^1^H NMR experiments. The ratios of *Z/E* isomers **4–7** were increased in the range of 10–60% in the VT ^1^H NMR experiments. The results of this study indicate that identifying the isomers of asymmetrical N-nitrosamine is necessary to control the quality of N-nitrosamines for active pharmaceutical ingredients (APIs).

## 1. Introduction

**Figure 1:**
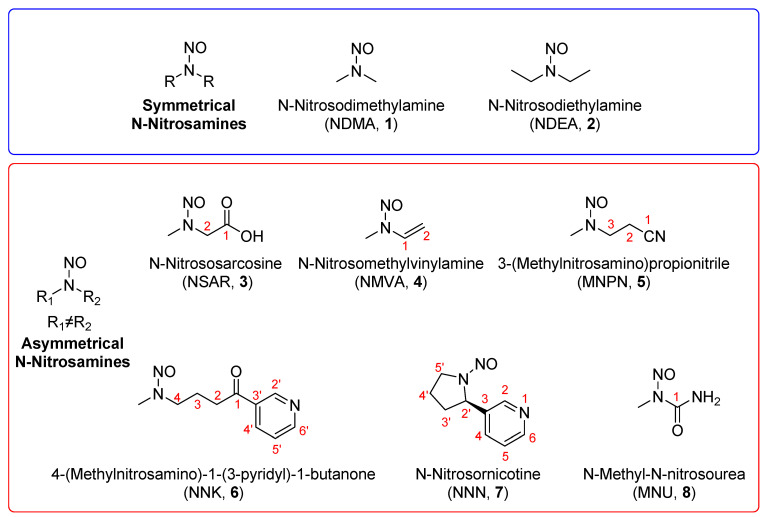
The chemical structures of some N-nitrosamines.

N-Nitrosamines are well-known pro-mutagens that can react with DNA following metabolism to produce DNA adducts, such as O^6^-alkyl-guanine. These adducts can result in DNA replication miscoding errors, leading to GC > AT mutations and an increased risk of genomic instability and carcinogenesis [1]. In 2018, N-nitrosodimethylamine (NDMA, **1**), a genotoxic carcinogen, was detected as a synthesis impurity in some valsartan drugs, while other N-nitrosamines, such as N-nitrosodiethylamine (NDEA, **2**), were later detected in other sartan products. In September 2019, the FDA stated that a low amount of NDMA had been detected in ranitidine. The FDA also announced that it had found excessive levels of NDMA in metformin in February 2022. Some N-nitrosamines, such as N-nitrososarcosine (NSAR, **3**), N-nitrosomethylvinylamine (**4**), 3-(methylnitrosamino)propionitrile (MNPN, **5**), 4-(methylnitrosamino)-1-(3-pyridyl)-1-butanone (NNK, **6**), N-nitrosornicotine (NNN, **7**) and N-methyl-N-nitrosourea (MNU, **8**), occur not only in drugs but also in pickled foods and tobacco (Figure 1). Therefore, controlling their concentrations in drugs, foods and tobacco is very important.

**Figure 2:**
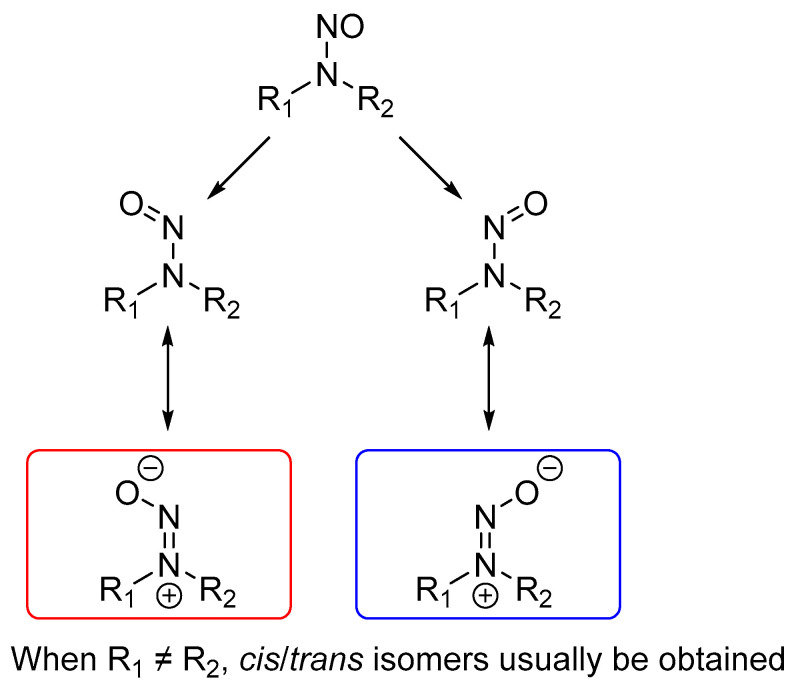
The possible mechanism of the generation of *Z*/*E* isomers of asymmetrical N-nitrosamines.

When we performed HPLC or GC analyses of certain asymmetrical N-nitrosamines, we often observed two peaks for one nitrosamine. This finding was attributed to the fact that asymmetrical N-nitrosamine may have configurational isomers due to the hindered rotation of a single bond (N‒N), resulting in strong variations in the anisotropic effects. The two conformers have features similar to those of the *E*/*Z* isomers relative to a double bond (Figure 2). A similar phenomenon has been reported, in which the stereospecific response of the *E/Z* isomers of NSAR (**3**) was determined by LC–ESI–MS/MS [2]. NSAR (**3**) and MNPN (**5**) have also been shown to produce two isomer peaks in the UPLC–MS/MS assay [3]. In this paper, we report a series of asymmetrical N-nitroamines (**3**–**7**) displaying two groups of NMR signals. However, few studies have reported the NMR assignment of asymmetrical N-nitroamines isomers. Inspired by the above phenomenon, variable-temperature (VT) ^1^H-NMR experiments were carried out to determine the percentage changes of the two configurational isomers, which revealed the configurational isomerism phenomenon. As density functional theory (DFT) calculations are widely used to determine NMR assignments for the characterization of complex structures [4,5], we performed DFT calculations to assign the NMR signals of these conformers. To our knowledge, this is the first report of the NMR assignment of configurational isomers of N-nitrosamines.

## 2. Results and Discussion

As shown in Figures S1–S12, the ^1^H-NMR spectrum of N-nitrososarcosine **3** showed one group of major signals (*δ*_H_ 3.79 and 4.28) and a set of minor signals (*δ*_H_ 3.01 and 5.01). In addition, the major carbon signals of **3** were observed at *δ*_C_ 40.0, 47.3 and 167.6, and the minor signals were observed at *δ*_H_ 33.0, 54.6 and 170.3. Similarly, the ^13^C-NMR spectrum of N-nitrososarcosine **4** showed two sets of different carbon signals. However, some of the ^1^H-NMR signals had differences, such as signals of ‒NCH_3_ (*δ*_H_ 3.15 vs. *δ*_H_ 3.89) and H-1 (*δ*_H_ 7.89 vs. *δ*_H_ 7.58). Two conformers of **5** showed two groups of distinct 1D NMR signals, of which the maximum difference in the ^1^H-NMR and ^13^C-NMR spectra between the two isomers was 0.77 ppm for ‒NCH_3_ (3.03 vs. 3.80 ppm) and 8.6 ppm for C-3 (49.1 vs. 40.5 ppm), respectively. Differences in the ^1^H-NMR spectra between the two isomers of **6** were present in the alkyl chain, including H-2, H-3 and H-4. Furthermore, the differences in their ^13^C-NMR spectra were associated with ‒NCH_3_ and the chain from C-2 to C-4. Two groups of NMR signals in the spectrum of **7** can be easily distinguished. Above all, the major and minor signals were also assigned based on the peak integration (Table 1 and Table 2). Furthermore, the configurational exchange and conformer ratios of **3**–**7** were investigated via a VT ^1^H-NMR experiment.

To further investigate the configurational behavior of asymmetrical N-nitrosamines **3**–**7**, DFT quantum chemical calculations were conducted [6]. Because the hindered rotation of the nitryl formed *E* and *Z* configurations, resembling the *Z*/*E* isomers relative to the double bond, two configurational isomers (a/b) were converted to *Z*/*E* for further calculations (Figure 2).

**Figure 3:**
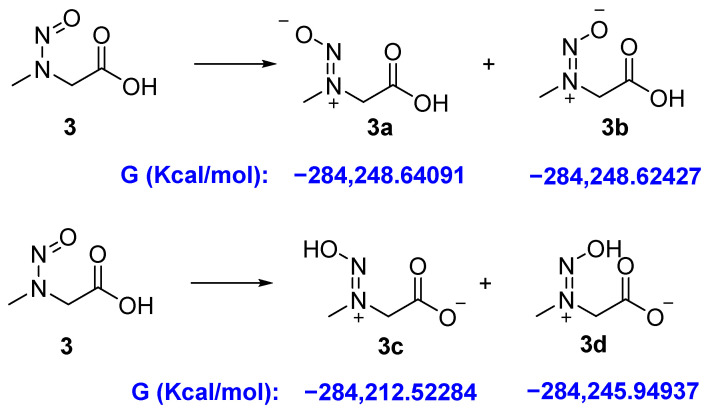
The Gibbs free energies and energy difference of the four possible conformers **3a**–**3d**.

Compound **3** may contain 4 isomers **3a**–**3d** (Figure 3). The DFT calculations showed that the Gibbs free energies of isomers **3c** and **3d** are higher than those of **3a** and **3b** (Figure 3), suggesting that they are more unstable than **3a** and **3b**; thus, we mainly considered the contributions of **3a** and **3b** to the NMR data.

**Figure 4:**
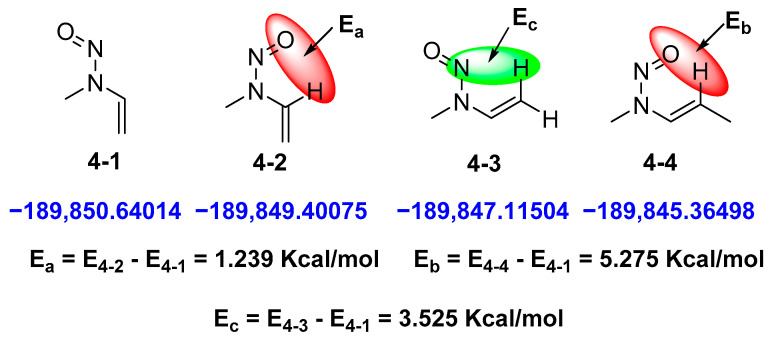
The Gibbs free energies and energy differences of four possible conformers for compound **4**.

Compounds **4** and **7** have *sp*^2^ CH or CH_2_, which could affect the stability of the isomers. For compound **4**, we considered four possible stable conformers, and their energies were calculated. As shown in Figure 4, the interaction of N=O and *sp*^2^ CH can be represented through the energy difference between E_1_ and E_2_. Similarly, the interaction between N=O and *sp*^2^ CH_2_ can be shown through the energy difference of E_4_–E_1_. In addition, the interaction between the nitrogen atoms of N=O and *sp*^2^ CH_2_ can be interpreted by the energy calculation of E_3_ and E_1_. On the basis of their energy differences, the closer *sp*^2^ values of CH or CH_2_ and N=O are, the more unstable they are. Thus, for compound **7**, the pyridine ring is rich in electrons, similar to the double bond in compound **4**, which repels the N=O-containing electrons. Meanwhile, considering the steric hindrance of pyridine, the *E*-configuration of **7** is more stable than the *Z*-configuration, which is consistent with the energy calculation.

**Figure 5:**
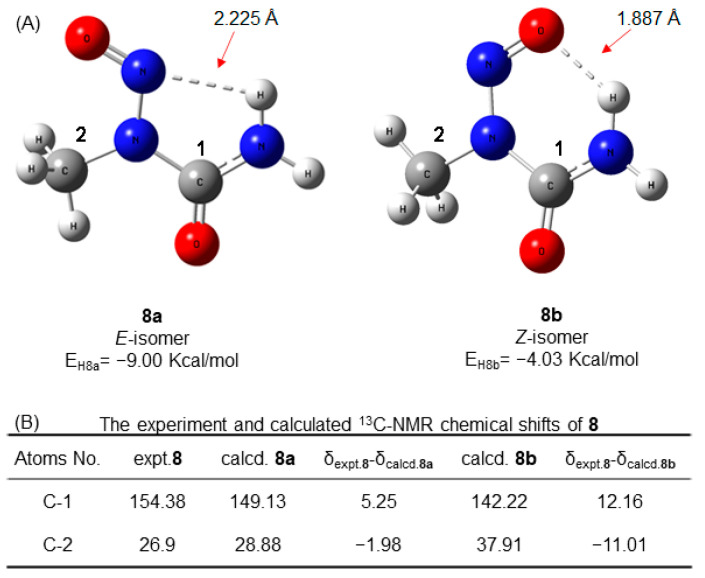
The possible conformers (**A**), their energy values (**A**) and the calculated ^13^C-NMR data (**B**) for N-nitrosamines of **8**.

Intriguingly, N-nitrososarcosine **8** showed only one set of NMR signals, suggesting that only one optimized conformer was present in **8**, which was caused by the key hydrogen bond between the oxygen or nitrogen in the nitryl moiety and the hydrogen in urea, restricting its configurational exchange. The presence of hydrogen bonds was established by energy calculations at the M062X/Def2TZVP level of theory. Both the *E* configuration (**8a**) and the *Z* configuration (**8b**) might form a hydrogen bond. The *E* configuration (**8a**) was predicted to be 4.97 Kcal/mol lower in energy than the *Z* configuration (**8b**), indicating that the *E* configuration (**8a**) may be the stable configuration, with an intermolecular hydrogen bond of approximately 2.225 Å (Figure 5A). The DFT quantum chemical calculations showed that the calculated ^13^C NMR data for the *E* configuration (**8a**) were less different from the experimental data. Based on the above evidence, one set of NMR signals was concluded to be from the *E* configuration (**8a**).

**Figure 6:**
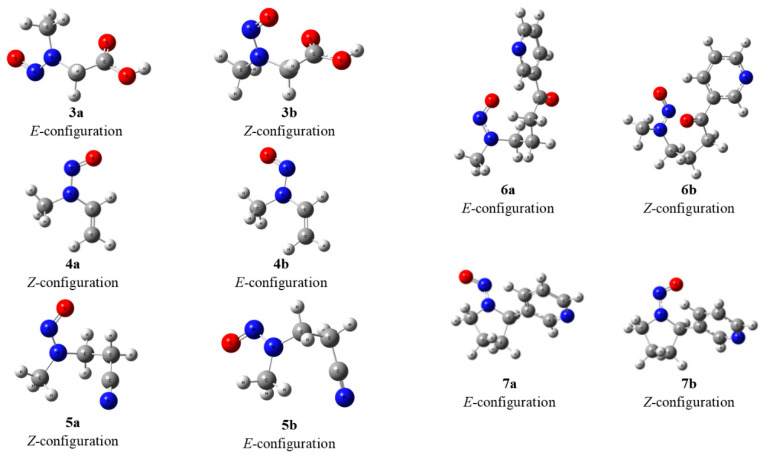
Optimized conformers derived from DFT calculations for asymmetrical N-nitrosamines **3**–**7**.

A summary of these calculated NMR data and their comparisons with experimental values are presented in Table 3 and Table 4, and the correlation coefficients are presented in Table 5; these data were used to assign the NMR signals for the *Z* and *E* configurations of N-nitrososarcosine **3**–**7**. Table 6 shows the Gibbs free energy values (G, Kcal/mol) of *Z*/*E* isomers for compounds **3**–**7** at the M062X/Def2TZVP level of theory with Grimme’s D3 correction. The major calculated molecular models of **3**–**7** are shown in Figure 6.

**Figure 7:**
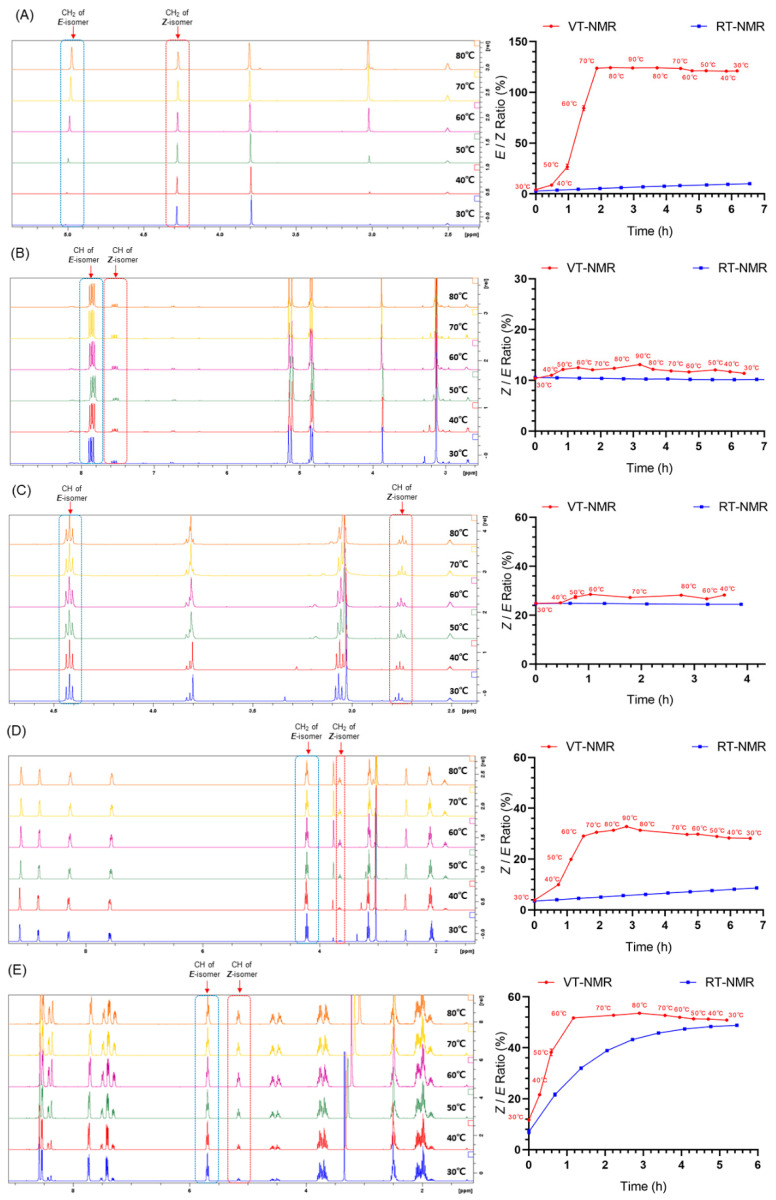
The *Z/E* ratios of asymmetrical N-nitrosamines **3**–**7** (**A**–**E**, respectively) at different temperatures.

To quantify the ratios of isomers and the changes in the ratio at different temperatures, we carried out VT NMR spectroscopic studies (Figure 7). All ratios of the **3**–**7** isomers were changed in the VT-NMR experiments. To determine whether these changes were affected by temperature or time, control NMR experiments were performed at room temperature (RT). Figure 7A shows that the *Z/E* ratio of **3** quickly increased in the VT-NMR experiment. This ratio was maintained at 120~130% even though the NMR probe temperature changed from 90 °C to 30 °C. In the control RT-NMR experiment of **3**, the *Z/E* ratio increased slowly from 2 to 12% within seven hours. A similar phenomenon was also observed in the VT/RT-NMR experiments of **6** (Figure 7D). The *Z/E* ratios of **4** and **5** exhibited small changes of approximately 12 and 24%, respectively (Figure 7B, C). In addition, **7** was shown to exhibit different changes in the *Z/E* ratios in the VT/RT-NMR experiment, but they ultimately showed a similar *Z/E* ratio of approximately 50%. Based on these VT/RT-NMR experiments, the rapid changes in the *Z/E* ratios of isomers **3**–**7** were temperature-dependent. To our surprise, when the NMR probe temperature returned to 30 °C from higher temperatures, the *Z/E* ratios did not show a significant decrease. This means there might be a balance between the two isomers in solvents.

## 3. Experimental Section

### 3.1. Materials and Reagents

N-Nitrososarcosine (NSAR, **3**), n-nitrosomethylvinylamine (**4**), 3-(methylnitrosamino)propionitrile (MNPN, **5**), 4-(methylnitrosamino)-1-(3-pyridyl)-1-butanone (NNK, **6**), N′-nitrosornicotine (NNN, **7**) and N-methyl-N-nitrosourea (MNU, **8**) were purchased from the Chemical Drug Control Institute of the China National Institutes for Food and Drug Control (NIFDC, Beijing, China). DMSO-*d6* with 0.03% tetramethylsilane (TMS) was purchased from Cambridge Isotope Laboratories.

### 3.2. NMR Experiments

NMR samples were prepared in DMSO-*d6* with 0.03% tetramethylsilane (TMS). The chemical shifts are quoted in ppm relative to TMS. ^1^H-NMR and ^13^C-NMR spectra were recorded on a 400 MHz Bruker NMR spectrometer (Bruker BioSpin GmbH, Ettlingen, Germany). VT ^1^H-NMR spectra were recorded at 30 °C, 40 °C, 50 °C, 60 °C, 70 °C, 80 °C and 90 °C. Before each sample was subjected to the VT ^1^H-NMR experiment, it was heated in an NMR probe at the experimental temperature (from 30 °C to 90 °C, then back to 30 °C) for at least 10 min. TopSpin 2.1 software (Bruker BioSpin, Billerica, MA, USA)was used for the acquisition and processing of the NMR data.

### 3.3. Computational Details

A conformational search was performed using Crest software (Loughborough University, Loughborough, The United Kingdom), and the conformers within an energy window of 5 kcal·mol^−1^ were optimized with DFT calculations at the M062X/Def2TZVP level of theory with Grimme’s D3 correction [7] using the Gaussian 09 program (Gaussian, Inc.: Wallingford, CT, USA) [8]. A frequency analysis was performed at the same level of theory to ensure that no imaginary frequencies existed and to determine the Gibbs free energies for the subsequent population analysis. Room-temperature (298.15 K) equilibrium populations were calculated according to the Boltzmann distribution law. Those conformers, accounting for over 99% of the population, were subjected to subsequent calculations.

The GIAO method [9,10,11,12,13] at the mPW1PW91/B3LYP/6–31+G(d, p) level of theory (in DMSO) in corresponding solvents with the IEFPCM solvent model [14] was used for the NMR calculation. The chemical shifts were calculated from shielding constants by referencing TMS at 0 ppm (*δ*_calcd_ = *σ*_TMS_ − *σ*_calcd_), where *σ*_TMS_ is the shielding constant of TMS calculated at the same level of theory. For each possible candidate, the parameters of the linear regression *δ*_cal_ = a × *δ*_exp_ + b and the correlation coefficient, *R*^2^, were determined.

The hydrogen bond energy (E_H_) was calculated using the equation E_H_ = E_8a_ − E_8_, where E_8a_ is the energy of the conformer without hydrogen bonding by twisting the N-N to break the hydrogen bond, and E_8_ is the energy of the optimized conformer [15].

## Figures and Tables

**Table 1:** ^1^H-NMR chemical shifts of asymmetrical N-nitrososarcosines **3**–**7**.

H Atmos	3		4		5		6		7	
No.	Major	Minor	Major	Minor	Major	Minor	Major	Minor	Major	Minor
NCH_3_	3.79	3.01	3.153	3.89	3.03	3.8	3.1	3.1		
1			7.89	7.58						
2	4.28	5.01	5.16	5.16	4.42	3.81	3.08	2.95	8.58	8.38
			4.86	4.87						
3					3.07	2.76	2.25	1.99		
4							4.27	3.8	7.73	7.51
5									7.41	7.33
6									8.53	8.43
2′							9.15	9.15	5.69	5.16
3′									2.5	1.84
4′							8.2	8.2	1.99	2.02
5′							7.4	7.4	3.67	4.48
6′							8.79	8.79		

**Table 2:** ^13^C-NMR chemical shifts of asymmetrical N-nitrososarcosine **3**–**7**.

C Atmos	3		4		5		6		7	
No.	Major	Minor	Major	Minor	Major	Minor	Major	Minor	Major	Minor
NCH_3_	40.0	33.0	27.4	36.6	31.6	39.4	31.5	39.0		
1	167.6	170.3	136.8	125.1	118.9	119.0	197.4	197.4		
2	47.3	54.6	98.9	101.3	17.1	14.3	35.1	35.7	148.9	147.9
3					49.1	40.5	21.7	19.9	137.3	136.3
4							52.8	43.8	135.0	133.7
5									124.2	123.9
6									149.4	148.5
2′							149.5	149.5	62.4	58.8
3′							131.8	131.8	33.5	33.3
4′							135.3	135.3	21.1	22.8
5′							123.7	123.7	46.7	51.0
6′							153.8	153.8		

**Table 3:** ^1^H NMR chemical shifts for asymmetrical N-nitrososarcosine **3**–**7** determined by DFT calculations.

H Atmos	3		4		5		6		7	
No.	3a	3b	4a	4b	5a	5b	6a	6b	7a	7b
NCH_3_	3.21	4.19	3.58	5.04	4.47	3.28	5.13	2.65		
	3.21	3.71	3.58	2.35	3.63	3.08	4.77	2.65		
	2.83	3.96	4.57	2.35	3.87	3.07	5.02	3.18		
1			8.04	8.19						
2	5.12	4.28	5.00	5.05	3.31	4.54	4.23	2.74	8.75	8.58
	5.12	4.02	5.17	5.19	4.11	4.31	3.98	3.08		
3					2.25	2.68	3.38	2.54		
					2.79	2.60	3.85	2.08		
4							5.01	3.87	7.56	7.57
							5.14	4.36		
5									7.59	7.52
6									8.73	8.66
2′							10.39	9.24	5.97	5.16
3′									2.53	2.39
									1.98	1.78
4′							9.69	8.23	1.90	2.14
									1.92	1.99
5′							8.92	7.69	3.62	4.56
									3.89	4.67
6′							10.06	8.91		

**Table 4:** ^13^C NMR chemical shifts for asymmetrical N-nitrososarcosine **3**–**7** determined by DFT calculations.

C Atmos	3		4		5		6		7	
No.	3a	3b	4a	4b	5a	5b	6a	6b	7a	7b
NCH_3_	35.8	40.9	37.3	25.0	41.5	36.0	39.7	35.4		
1	168.7	165.4	121.5	136.1	117.8	117.3	198.0	199.6		
2	56.3	49.6	97.0	95.6	16.5	21.3	35.1	36.8	146.5	146.1
3					44.2	52.0	24.9	27.0	137.8	135.8
4							44.3	56.0	131.6	131.1
5									121.3	121.3
6									147.1	146.7
2′							149.4	148.9	65.3	62.6
3′							128.5	130.0	36.5	36.0
4′							135.2	135.2	22.3	25.0
5′							121.4	121.2	49.1	53.0
6′							152.4	152.4		

**Table 5:** Correlation coefficients of the calculated and experimental ^13^C-NMR chemical shifts for N-nitrososarcosine **3**–**7**.

Calculated Conformers	R^2^ of Conformers		Conclusion
	Major	Minor	
*E*-**3a**	0.995686	**0.999995**	Major conformer of **3** is *Z*
*Z-* **3b**	**0.999931**	0.996440	
*Z-* **4a**	0.998206	**0.999687**	Major conformer of **4** is *E*
*E*-**4b**	**0.999783**	0.994411	
*Z-* **5a**	0.990292	**0.999707**	Major conformer of **5** is *E*
*E*-**5b**	**0.999939**	0.989404	
*Z-* **6a**	0.997660	**0.999497**	Major conformer of **6** is *E*
*E*-**6b**	**0.999468**	0.997347	
*E*-**7a**	**0.999590**	0.998584	Major conformer of **7** is *E*
*Z-* **7b**	0.999464	**0.999750**	

**Table 6:** The Gibbs free energy values (G, Kcal/mol) of *Z*/*E* isomers for compounds **3**–**7** at the M062X/Def2TZVP level of theory with Grimme’s D3 correction.

Compounds	*Z*	*E*	Difference Value
**3**	−284,248.62427	−284,248.64091	0.017
**4**	−189,849.40075	−189,850.64014	1.239
**5**	−248,452.662324	−248,452.232420	0.430
**6**	−441,336.708517	−441,336.80758	0.099
**7**	−369,479.165009	−369,479.778283	0.613

## Data Availability

The data presented in this study are contained within the article and supplementary materials.

## Supplementary Materials

The following supporting information can be downloaded at: https://www.mdpi.com/article/10.3390/molecules27154749/s1. Figure S1: ^1^H NMR spectrum of compound **3**. Figure S2: ^13^C NMR spectrum of compound **3**. Figure S3: ^1^H NMR spectrum of compound **4**. Figure S4: ^13^C NMR spectrum of compound **4**. Figure S5: ^1^H NMR spectrum of compound **5**. Figure S6: ^13^C NMR spectrum of compound **5**. Figure S7: ^1^H NMR spectrum of compound **6**. Figure S8: ^13^C NMR spectrum of compound **6**. Figure S9: ^1^H NMR spectrum of compound **7**. Figure S10: ^13^C NMR spectrum of compound **7**. Figure S11: ^1^H NMR spectrum of compound **8**. Figure S12: ^13^C NMR spectrum of compound **8**.
