# Supplementary material for: NMR Assignments of Six Asymmetrical N-Nitrosamine Isomers Determined in an Active Pharmaceutical Ingredient by DFT Calculations
Source: Molecules. 2022 Jul 25;27(15):4749. doi: 10.3390/molecules27154749 (PMC9331877; doi:10.3390/molecules27154749)
Supplement: Supplementary file 1 [file molecules-27-04749-s001.zip › molecules-1780394-supplementary.pdf]

Supporting Information

NMR Assignments of Six Asymmetrical N-Nitrosamine Isomers Determined by DFT Calculations

Hao-Yue Guan, Yu-Fei Feng，Bai-Hao Sun, Jian-Zhao Niu*, Qing-Sheng Zhang*

Chemical drugs control institute of China National Institutes for Food and Drug Control (NIFDC), No.2 Tian Tan Xi Li Street, Dong Cheng District, Beijing 100050，China

*Corresponding author

E-mail: zqs@nifdc.org.cn (Qing-sheng Zhang); njz@nifdc.org.cn (Jian-Zhao Niu)

List of contents

1. Figure S1 ^{1}H NMR spectrum of compound **3**
2. Figure S2 ^{13}C NMR spectrum of compound **3**
3. Figure S3 ^{1}H NMR spectrum of compound **4**
4. Figure S4 ^{13}C NMR spectrum of compound **4**
5. Figure S5 ^{1}H NMR spectrum of compound **5**
6. Figure S6 ^{13}C NMR spectrum of compound **5**
7. Figure S7 ^{1}H NMR spectrum of compound **6**
8. Figure S8 ^{13}C NMR spectrum of compound **6**
9. Figure S9 ^{1}H NMR spectrum of compound **7**
10. Figure S10 ^{13}C NMR spectrum of compound **7**
11. Figure S11 ^{1}H NMR spectrum of compound **8**
12. Figure S12 ^{13}C NMR spectrum of compound **8**

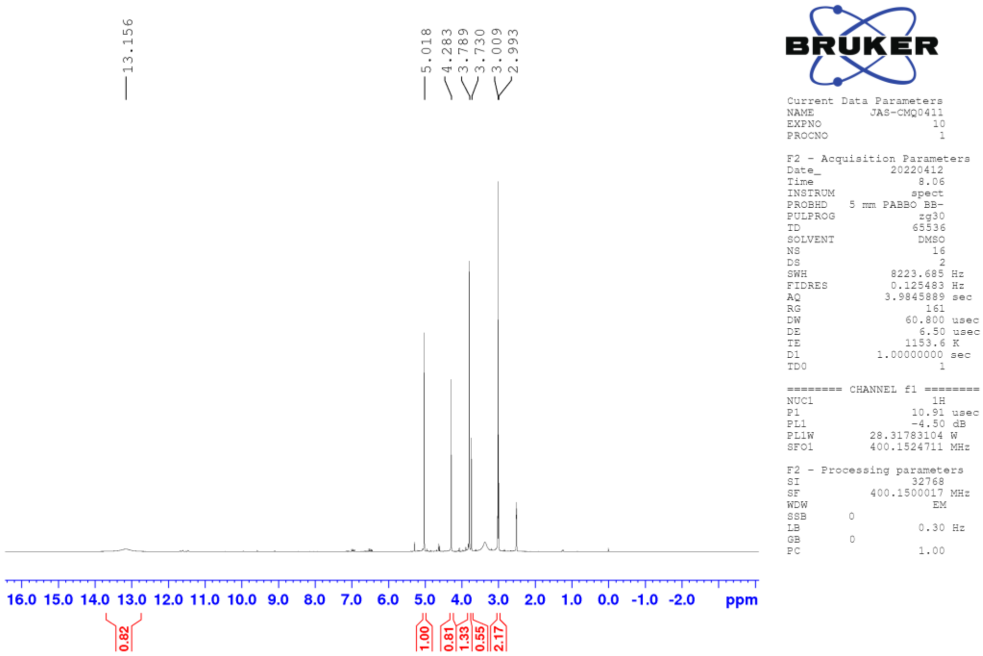

Figure S1 ^{1}H NMR spectrum of compound **3**

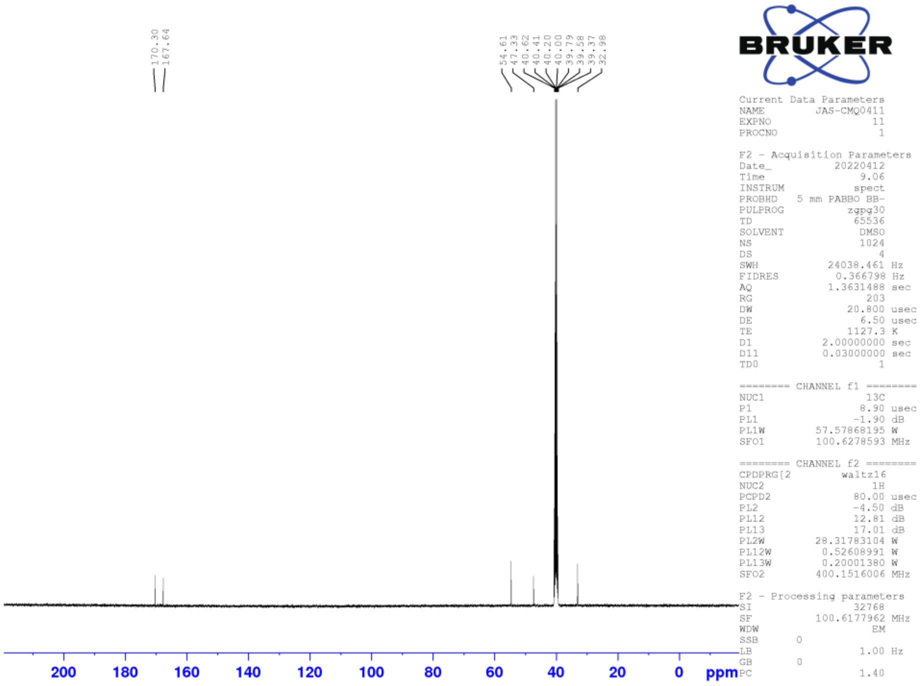

Figure S2 ^{13}C NMR spectrum of compound **3**

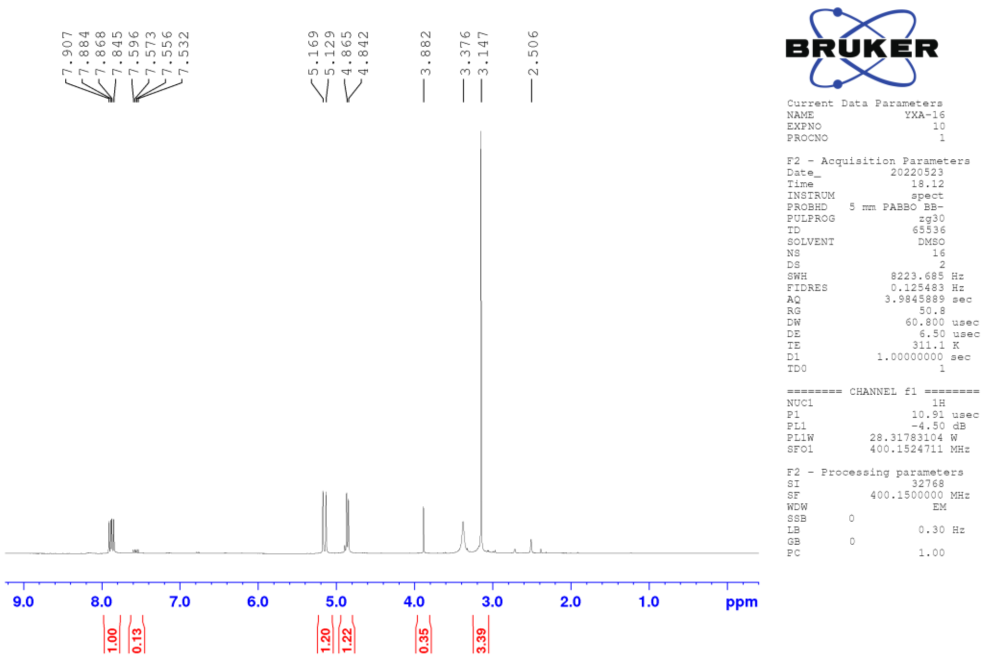

Figure S3 ^{1}H NMR spectrum of compound **4**

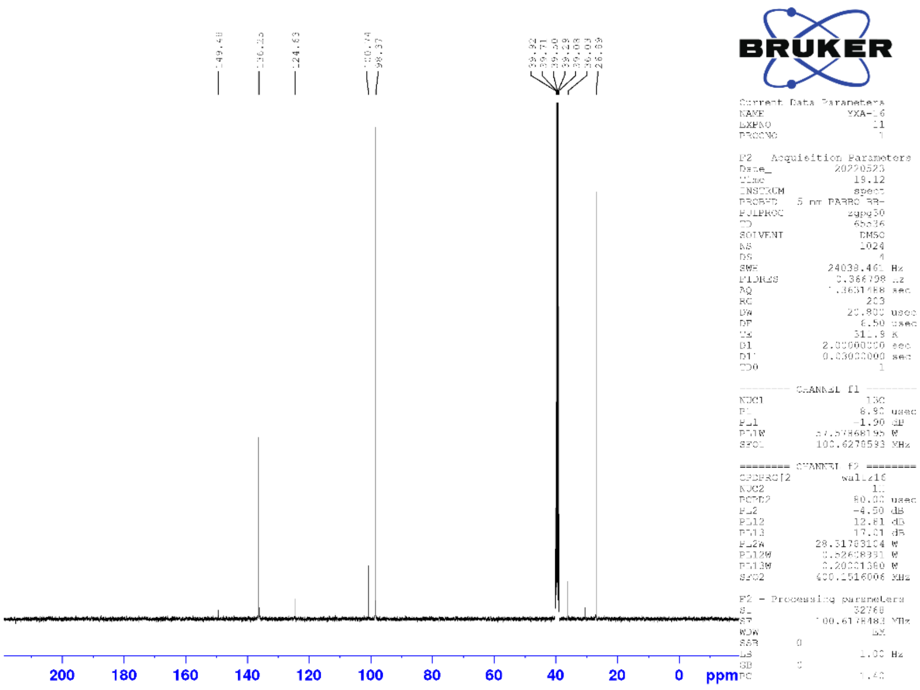

Figure S4 ^{13}C NMR spectrum of compound **4**

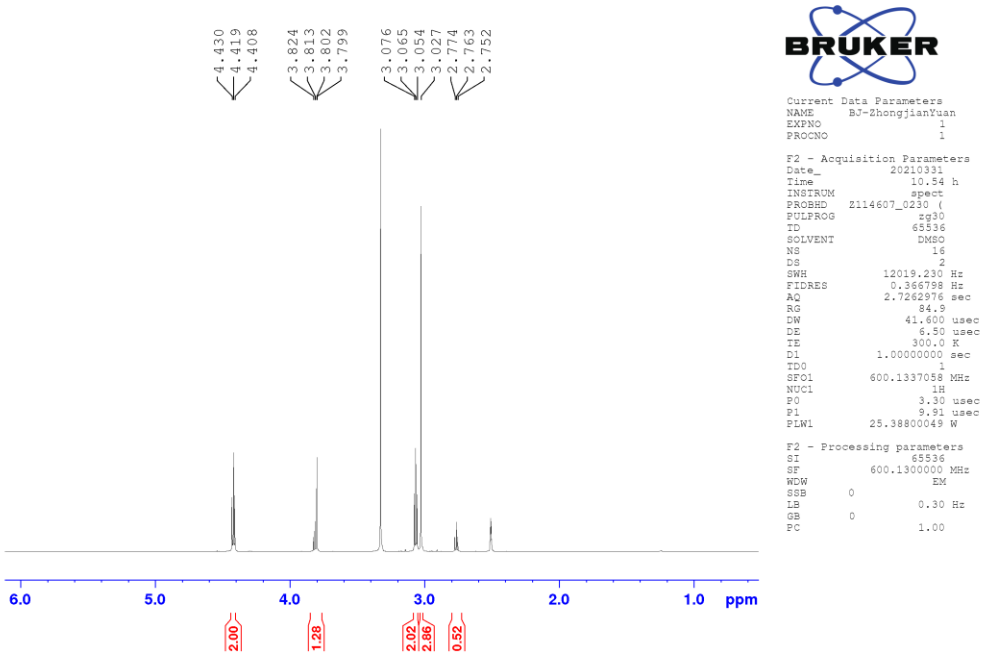

Figure S5 ^{1}H NMR spectrum of compound **5**

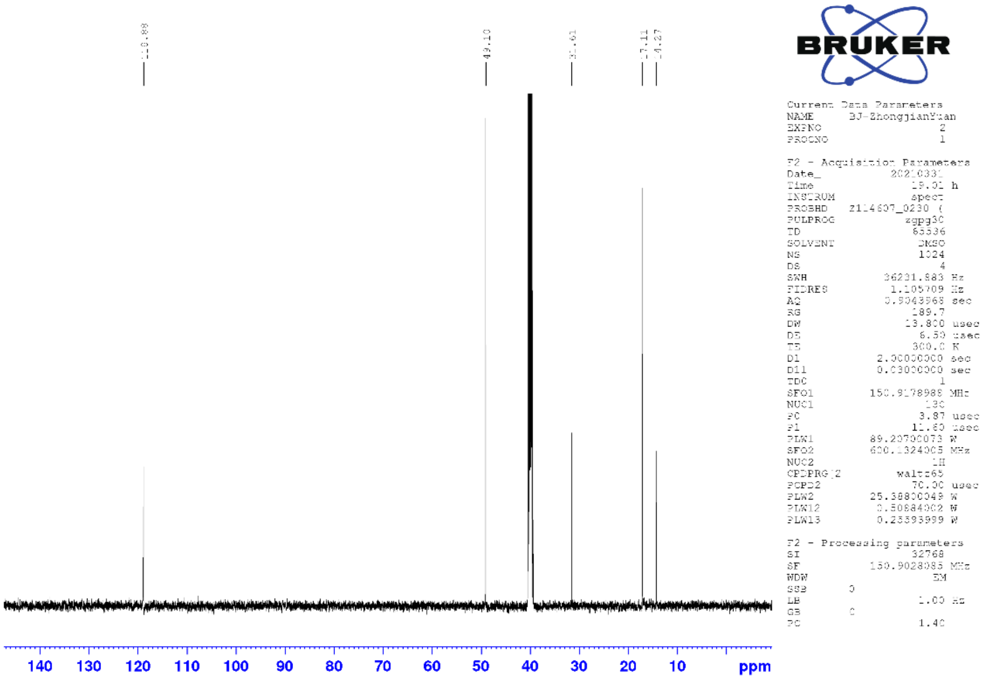

Figure S6 ^{13}C NMR spectrum of compound **5**

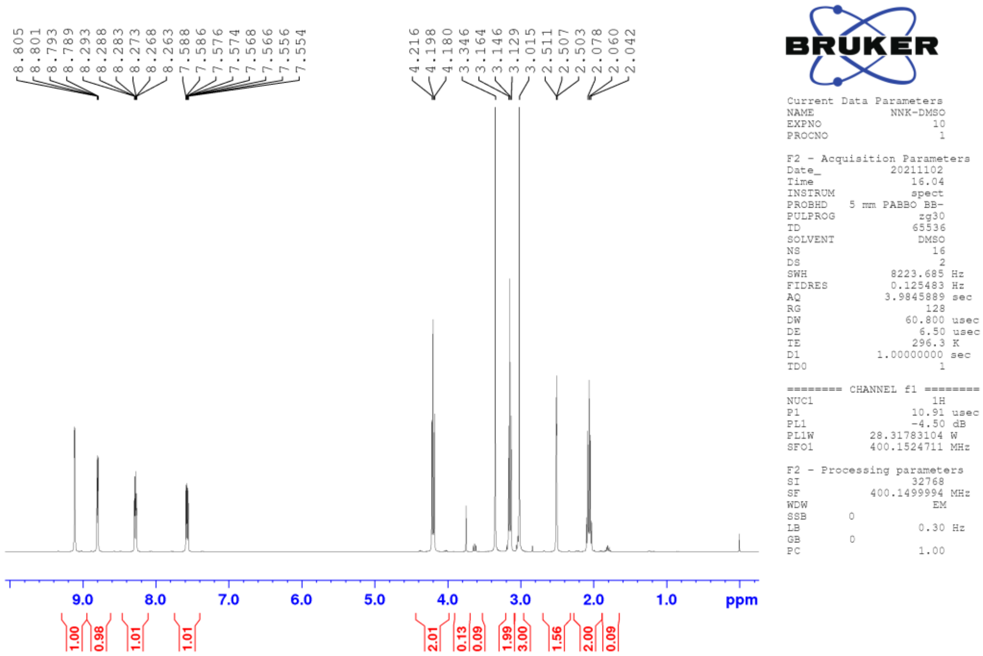

Figure S7 ^{1}H NMR spectrum of compound **6**

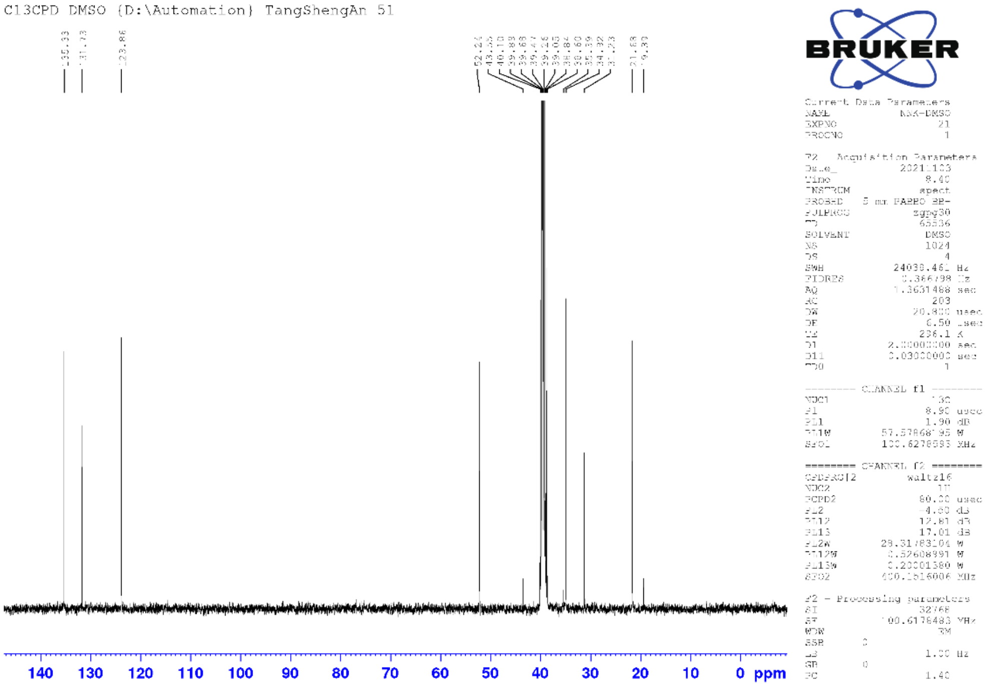

Figure S8 ^{13}C NMR spectrum of compound **6**

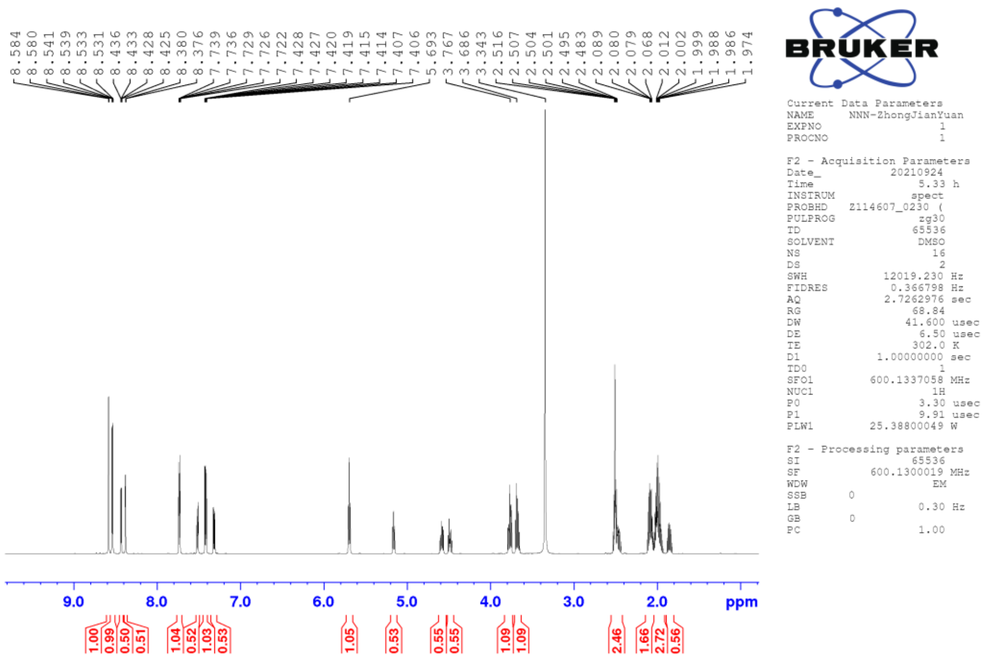

Figure S9 ^{1}H NMR spectrum of compound **7**

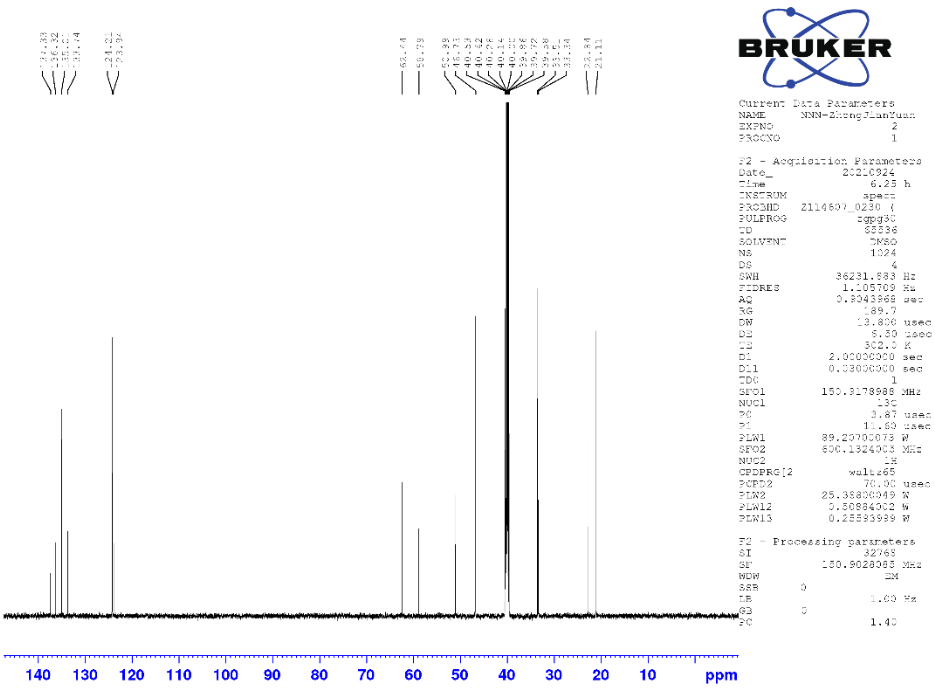

Figure S10 ^{13}C NMR spectrum of compound **7**

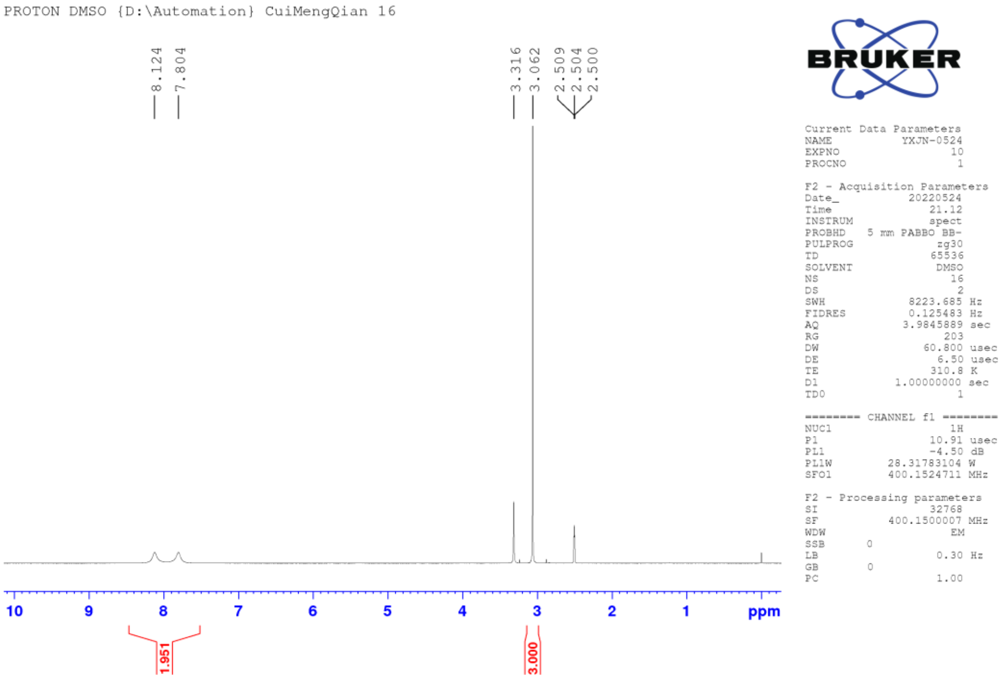

Figure S11 ^{1}H NMR spectrum of compound **8**

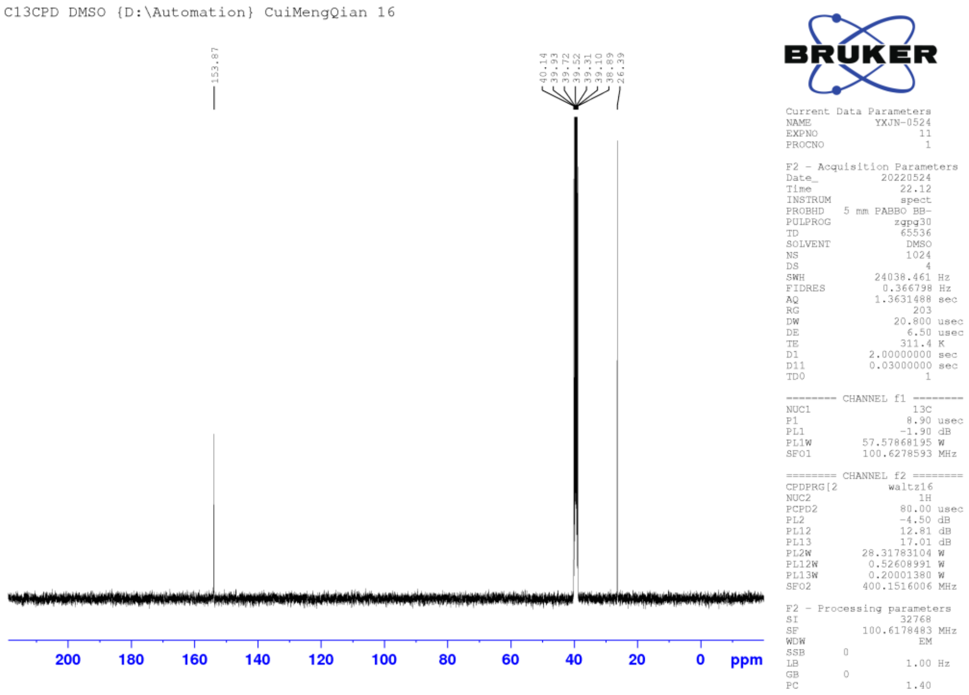

Figure S12 ^{13}C NMR spectrum of compound **8**
